# Parameters Influencing PET Imaging Features: A Phantom Study with Irregular and Heterogeneous Synthetic Lesions

**DOI:** 10.1155/2018/5324517

**Published:** 2018-09-10

**Authors:** Francesca Gallivanone, Matteo Interlenghi, Daniela D'Ambrosio, Giuseppe Trifirò, Isabella Castiglioni

**Affiliations:** ^1^Institute of Molecular Bioimaging and Physiology, National Research Council (IBFM-CNR), Milan, Italy; ^2^Medical Physics Unit, IRCCS Fondazione S. Maugeri, Pavia, Italy; ^3^Nuclear Medicine Unit, IRCCS Fondazione S. Maugeri, Pavia, Italy

## Abstract

**Aim:**

To evaluate reproducibility and stability of radiomic features as effects of the use of different volume segmentation methods and reconstruction settings. The potential of radiomics in really capturing the presence of heterogeneous tumor uptake and irregular shape was also investigated.

**Materials and Methods:**

An anthropomorphic phantom miming real clinical situations including synthetic lesions with irregular shape and nonuniform radiotracer uptake was used. ^18^F-FDG PET/CT measurements of the phantom were performed including 38 lesions of different shape, size, lesion-to-background ratio, and radiotracer uptake distribution. Different reconstruction parameters and segmentation methods were considered. COVs were calculated to quantify feature variations over the different reconstruction settings. Friedman test was applied to the values of the radiomic features obtained for the considered segmentation approaches. Two sets of test-retest measurement were acquired and the pairwise intraclass correlation coefficient was calculated. Fifty-eight morphological and statistical features were extracted from the segmented lesion volumes. A Mann–Whitney test was used to evaluate significant differences among each feature when calculated from heterogeneous versus homogeneous uptake. The significance of each radiomic feature in terms of capturing heterogeneity was evaluated also by testing correlation with gold standard indexes of heterogeneity and sphericity.

**Results:**

The choice of the segmentation method has a strong impact on the stability of radiomic features (less than 20% can be considered stable features). Reconstruction affects the estimate of radiomic features (only 26% are stable). Thirty-one radiomic features (53%) resulted to be reproducible, 11 of them are able to discriminate heterogeneity. Among these, we found a subset of 3 radiomic features strongly correlated with GS heterogeneity index that can be suggested as good features for retrospective evaluations.

## 1. Introduction

From its introduction in clinical practice, medical imaging has gained a central role in the management of a large variety of diseases. In particular, in oncology, medical imaging shows its unique property of characterizing, in vivo and noninvasively, the onset and progression of pathological processes at different stages of diseases [1].

In clinical practice, at a first level, medical images are qualitatively inspected by radiologists or nuclear medicine physicians [2]. However, such qualitative analysis presents several limitations: a certain level of subjectivity that can cause a lack of standardization in the assessment as well as problems in some follow up evaluations.

To overcome such limitations, a great effort was focused in the recent years to develop quantitative approaches to medical image analysis. These approaches exploit the fact that digital medical images are inherently quantitative and that their quantitative values express several tissue functional characteristics, such as metabolism or proliferation [3], with a role for the onset and progression of cancer. Recent studies have been devoted to the development of automatic or semiautomatic methods for the extraction of quantitative indexes from images to be used as imaging biomarkers of cancer disease [4, 5].

Thanks to the advancements in such image processing methods, macroscopic indexes such as the Standardized Uptake Value (SUV) for Positron Emission Tomography (PET) or the Apparent Diffusion Coefficient (ADC) for Magnetic Resonance Imaging (MRI), and measuring global functional properties of an oncological lesion, were proven effective biomarkers for diagnosis or treatment response in oncological clinical studies [6, 7]. However, novel quantitative features have been more recently explored to capture regional characteristics of a cancer lesion not always perceivable to the naked eye, such as inter- and intratumor heterogeneity, that may have an impact on the clinical outcome of different cancer phenotypes [8]. The rationale behind such advanced image features is the hypothesis that imaging *in vivo* heterogeneities of a cancer lesion is able to reflect the tumor phenotype with the advantage of a noninvasive technique. The recent literature shows the high potential of such quantitative heterogeneity features, defined “radiomics” [5], thanks to their proven abilities to be correlated with “omics” data. Radiomics refer to a large number of mathematical image descriptors extracted from the volume of an entire cancer lesion by the use of different image analytics methods, including morphological and statistical analyses [8].

Promising results published in several increasing papers proved that radiomic traits reflect tumor heterogeneity which is correlated to bad prognosis [9]. However, few studies were performed to evaluate how really those radiomic features are related to the actual shape or tissue heterogeneity of the tumor [10].

Furthermore, from a methodological point of view, one of the key problems emerging when defining image quantitative features is to assess their reproducibility, which is the closeness of the agreement between the results of successive measurements of the features carried out under the same conditions of measurement.

Moreover, different measurement conditions, such as different image reconstruction settings or lesion volume segmentation methods, can highly impact on the image feature stability, posing serious issues on the use of some image features as disease biomarkers [11–13].

The main purpose of this work is to evaluate reproducibility and stability of some radiomic features as effects of the use of different volume segmentation methods and reconstruction settings, which currently represent the more common variables in retrospective clinical oncological studies. We then assessed the significance of such radiomic features in effectively characterizing the lesion heterogeneity and shape.

These aims were pursued with the use of a realistic dataset of PET images obtaining from a thorax anthropomorphic phantom miming realistic oncological lesions with irregular shape and heterogeneous uptake of radiotracer whose GSs were known. Our work is helpful in determining the limits and the quantitative properties for clinical application of the radiomics approach with respect to the tested methods and parameters.

## 2. Materials and Methods

### 2.1. Phantom Setting and PET Data Acquisition

The anthropomorphic Alderson Thorax phantom (Radiology Support Devices, Inc.) was used to simulate man/woman thorax or breast body districts. Several synthetic lesions of irregular shape and both homogeneous and heterogeneous uptakes were realized and placed inside the thorax or the breasts of the anthropomorphic phantom within ^18^F-FDG radioactive background. In order to simulate realistic patient PET studies, each phantom compartment was filled with a different background of ^18^F-FDG radioactivity concentration: lungs with 0.004 MBq/cc, liver with 0.013 MBq/cc, myocardial wall with 0.023 MBq/cc, thorax with 0.006–0.007 MBq/cc, and breasts with 0.002–0.009 MBq/cc [14].

The whole procedure of preparing the phantom before PET acquisitions took about two hours. The ^18^F-FDG radioactivity concentration used during the preparation of the phantom took into account this time frame and was recalculated based on the half life of the ^18^F-FDG.

A strategy to produce realistic oncological lesions of irregular shape with a homogeneous or a heterogeneous uptake of ^18^F-FDG [15] was adopted by using 3D-printed irregular shells filled with different concentrations of radioactive gels.

To obtain realistic oncological lesions with irregular shape, we defined 3D shells by segmenting the lesion volumes of different oncological lesions on ^18^F-FDG PET/CT images of real patients. The segmented volumes were then processed in order to generate images of 3D surfaces of lesions, saved in digital files. These surfaces were then cut into two parts by image manipulation and 3D printed using a 3D printer (Renkforce RF1000 Single Extruder) equipped with plastic filaments of 3 mm diameter (Renkforce PLA300 Plastic PLA 3 mm), thus manufacturing plastic moulds of patient-derived oncological lesions.

The availability of the printed shells allowed obtaining the gold standard (GS) for the sphericity of the shells to be compared with geometrical characteristics of radiomic features as extracted from the PET images of the experimental studies performed with the phantom. In particular, for each printed mould, an index of sphericity was defined as the ratio between the surface of the sphere, with volume equivalent to actual mould volume (*V*_m_) and the actual shell surface (*S*_m_) of the mould.

(1) $$\begin{array}{c} S_{\text{GS}}=\frac{\pi^{1/3}{\left(6V_{m}\right)}^{2/3}}{S_{m}}. \end{array}$$

This index ranges from 0 to 1, where S_GS_ = 1 expresses a full spherical shape.

For the PET experimental measurements, the shells were filled with a radioactive gel produced with a fast-setting, chromatic, dust-free alginate powder (phase plus, Zhermack Clinical SpA–Badia Polesine (RO), Italy) mixed with a water solution of ^18^F-FDG [4]. Lesions with a uniform radioactive uptake were simulated using a gel preparation at a single radioactivity concentration, while gels obtained at different ^18^F-FDG concentrations were used for lesions simulating heterogeneous uptake.

**Figure 1:**
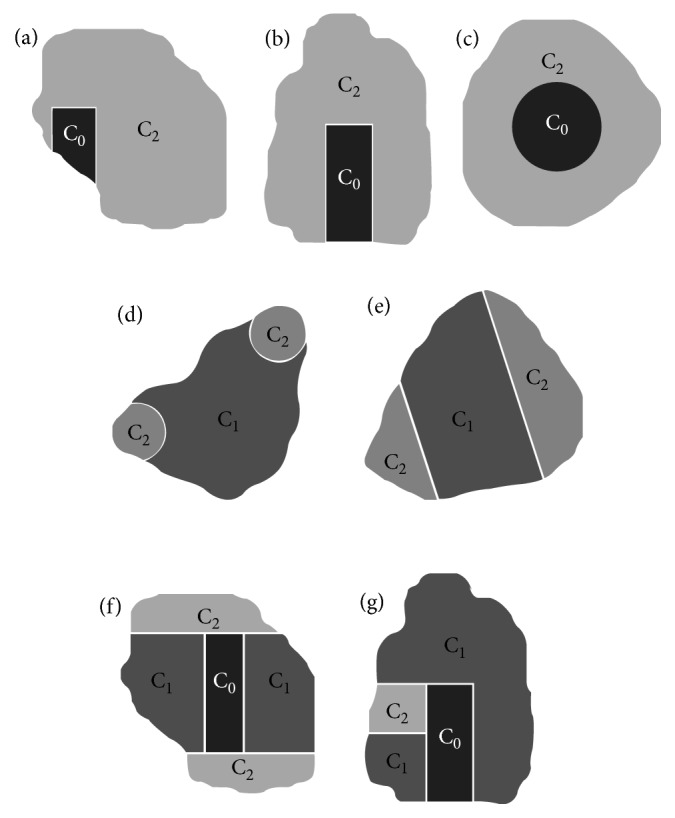
The seven different configurations to obtain lesions with different heterogeneous uptake. C_1_, C_2_, and C_0_ represent areas with lower, higher, and no radioactivity concentration, respectively. (a–c) Strategies for reproducing necrotic tissue; (d, e) heterogeneous (multifocal) uptake; (f, g) heterogeneous uptake and necrotic tissue.

Seven experimental configurations were studied, with different radioactivity concentrations (*C*_0_ = 0 MBq/cc, *C*_2_ = 5 ∗ C_1,_ C_1_ ranging from 0.03 to 0.16 MBq/cc). We thus obtained realistic oncological lesions with heterogeneous uptake, including necrotic tissues or multifocal uptake (Figure 1).

GSs for the lesion volumes (*V*_GS_) and the radioactivity concentrations were easily obtained for both homogeneous and heterogeneous lesions and the gel density and the net shell weight were found by using an analytical balance and a gamma counter (PerkinElmer 1480-011 Wizard 3”). In particular, in the case of heterogeneous lesions, for each filling with gels at different radioactivity concentrations, gel weights were obtained by the exact weight estimation of gel contributions at different radioactivity concentrations.

To obtain GSs for assessing the heterogeneity significance of radiomic features (as extracted from the PET studies of the phantom), two different indices of heterogeneity were considered.

The coefficient of variation of the different gels was measured as an index of heterogeneity in the radioactivity uptake, defined as the percentage ratio between the standard deviation and the mean of the radioactivity concentration within the lesion volume (COV_GS_).

The Gini index [16] was used to quantify the impact of spatial distribution of the uptake within the shell volume (*I*_G-GS_), has values from 0 in case of minimal heterogeneity to 1 in case of maximal heterogeneity, and was defined by processing digital files of shell surfaces used to print the shells. Generally, this index measures the heterogeneity of a statistical distribution in terms of the relative frequencies of the different modalities of a statistical variable. For each shell, each voxel of the corresponding shell image was considered as a statistical unit. For each voxel, the different modalities were defined as the different radioactivity concentrations used to fill the shell. Relative frequencies of each modality were calculated as the percentage of voxels occupied by each distinct radioactivity concentration.

The product of COV_GS_ and *I*_G-GS_ was considered as a GS index of total heterogeneity (*H*_GS_), with values from 0 to 100 for lower to higher heterogeneity.

The GS for lesion-to-background ratio (*L*/*B*_GS_) of each lesion was evaluated by measuring by the gamma counter the radioactive background of each phantom compartment where the lesions were arranged (breast containers and thorax).

^18^F-FDG PET-CT phantom measurements were performed on a Discovery 690 PET/CT system (General Electric Medical Systems) [17]. Each PET study had an acquisition time of 180 sec for each bed position (two bed positions for each PET acquisition). Image noise was also evaluated as COV of uptake distribution.

Images were reconstructed with a standard protocol optimized for whole-body clinical oncological studies: ordered subset expectation maximization (OSEM) in 3D mode, including Point Spread Function (PSF) [18–21] and Time of Flight modelling (TOF) [19–22], 3 iterations and 18 subsets, 5 mm filter cut-off and standard *z* axis filter, reconstructed matrix size 256 × 256, and transaxial field-of-view of 70 cm.

### 2.2. Image Segmentation

PET images of lesions were segmented in order to obtain the Metabolic Tumor Volume (MTV) from which extract the radiomic features. Segmentation methods used in this work included an adaptive threshold method and a fixed threshold method. The adaptive method was calibrated and validated on a variety of synthetic lesions miming real oncological lesions (i.e., with spherical and nonspherical shape and with homogenous and nonhomogenous ^18^F-FDG uptake), with an accuracy in the MTV measurement of 92% [4]. The fixed threshold method was implemented by using a cut-off of 60% from the maximum lesion uptake value. This threshold found a good compromise between a good estimate of the lesion volume and a good estimate of the lesion uptake, minimizing the possibility to include radioactivity background in the estimate [20, 21]. The two segmentation methods were implemented using Matlab and included in home-made software [22].

Since it has been shown that the use of thresholding approaches is appropriate for small lesions only when there is a good *L*/*B*, we calculated the percent error on the MTV estimate as a function of *L*/*B* for lesions with Volume GS < 10 cc, excluding from this computation the volume of necrotic regions (*C*_0 _= 0) when present within a lesion.

### 2.3. Radiomic Feature Extraction

Radiomic imaging features were extracted from each segmented MTV as morphological and statistical imaging features. Morphological imaging features (IF_M_) were obtained starting from the shape and size characteristics of the segmented MTV [5, 8].

The statistical analysis of first-order histogram describing the distribution of voxel intensities in MTV enabled to extract first-order statistical imaging features (IF_HIST_).

Texture analysis allowed obtaining statistical imaging features of higher orders. Images were resampled with an isotropic voxel size, considering the axial image size as resampled size. The MTV content was then resampled in 64 discrete gray-level values, and the texture analysis was performed with an in‐house‐developed MATLAB routine (v.2015b, MathWorks, Natick, MA, USA), largely based on a publicly available code [22]. Textural features were obtained from the analysis of the gray-level co-occurrence matrix (IF_TX-GLCM_), the gray-level run-length matrix (IF_TX-GLRLM_), the gray-level size zone matrix (IF_TX-GLSZM_), and the neighborhood gray tone difference matrix (IF_TX-NGTDM_). These matrices were obtained by the analysis of MTVs with 26-voxel connectivity, considering all possible direct connectivity with voxels in the same slice (8) and in the two adjacent slices (9 + 9 = 18).

### 2.4. Stability of Radiomic Features vs. Segmentation

In order to evaluate the impact of lesion volume segmentation (MTV) on the stability of radiomic features, the Friedman test was applied to the values of the radiomic features obtained for the two considered segmentation approaches (adaptive and fixed threshold methods, Section “Image Segmentation”).

### 2.5. Stability of Radiomic Features vs. Reconstruction

To study the impact of reconstruction settings on stability of radiomic features, PET images were reconstructed with reconstruction algorithms or parameters different with respect to the standard reconstruction protocols (section “Phantom setting and PET data acquisition”). For each reconstruction setting, lesions MTVs were extracted with the adaptive threshold segmentation method.

Reconstructions were performed with OSEM with or without PSF modelling and considering or omitting TOF. The impact of the matrix size of reconstructed images was also evaluated. Considering algorithm parameters, the influence of the number of iterations and subsets was assessed fixing a matrix size equal to 256 × 256, because it is the most used size in clinical practice. In order to evaluate the impact of the full width at half maximum (FWHM) of Gaussian filter, matrix size was chosen such that the reconstructed voxel size is within 3.0–4.0 mm in any direction and FWHM not exceeding 7 mm, according to EANM guidelines [23].

Table 1 lists reconstruction algorithms and parameters used and their impact evaluated in this work.

For each radiomic feature, COV were calculated as average of all lesions to quantify variations over the different reconstruction settings, thus characterizing feature stability vs. reconstruction.

On the basis of COV results, radiomic features were categorized into 4 groups: stable (COV ≤ 5%), quite stable (5% < COV ≤ 10%), poorly stable (10% < COV ≤ 20%), and unstable (COV > 20%).

For each feature, in order to provide representative information on its stability with respect to the different explored reconstruction settings, we considered the higher value of COVs obtained among all the reconstruction settings. A feature was considered quite stable when such COV value was found ≤10%.

### 2.6. Reproducibility of Radiomic Features

In order to explore reproducibility of radiomic features, a test-retest setting was used. Two sets of test-retest images were acquired approximately 30 min apart (acquisition time of 180 sec for each bed position). Lesions in the two sets of test-retest images were segmented with the adaptive threshold segmentation method.

For each feature, the pairwise intraclass correlation coefficient (ICC) was calculated [15]. Each feature with ICC > 0.6 in both of the two test-retest datasets (good or excellent agreement) was considered as a stable feature in test-retest setting.

### 2.7. Evaluation of Significance of Radiomic Features

A Mann–Whitney test was used to evaluate significant differences among each feature when calculated from heterogeneous vs. homogeneous uptake, thus measuring the potential of radiomic features in discriminating heterogeneous from homogeneous lesions.

The significance of each radiomic features in terms of capturing heterogeneity was evaluated also by testing correlation of each feature with *H*_GS_.

The morphological radiomic feature “Sphericity” was evaluated in its ability to reflect geometrical characteristics as defined by *S*_GS,_ using a paired *t*-test. Heterogeneous lesions with a necrosis inside were excluded from the analysis since it was difficult to define the surface of active component of lesion.

## 3. Results

### 3.1. Phantom Setting and PET Data Acquisition

Table 2 reports GS values for each ^18^F-FDG PET/CT acquisition.

Nine ^18^F-FDG PET/CT acquisitions of the phantom have been performed including 38 lesions of different shape, size, radiotracer distribution, and L/B ratio in different locations of the phantom. Five different 3D-printed shells with irregular shape (A-E) were used as obtained from the PET image segmentation of real oncological lesions. Their *V*_GS_ ranged from 6.8 to 32.3 cc. Their sphericity index, *S*_GS_, ranged from 0.49 to 0.74.

20/38 lesions were prepared with a uniform radiotracer uptake, while the remaining 18 lesions with a heterogeneous uptake. The *H*_GS_ for the 18 lesions with heterogeneous uptake ranged from 12.7 to 62.2, for lower to higher differences in radioactive uptake and its spatial distribution. In particular, their *V*_GS_ ranged from 6.4 to 29.4 cc (excluding from this computation the volume of necrotic regions when present within a lesion).

Explored L/B_GS_ ranged from 4 to 27.

*L*/*B* measured (*L*/*B*_m_) on PET images by using a validated PET quantification technique [24] ranged from 4.3 to 27.6.

Image noise of each PET acquisition was evaluated as COV in uptake distribution inside a large region of the liver. The mean COV calculated on the 9 PET acquisition is <8%.

**Figure 2:**
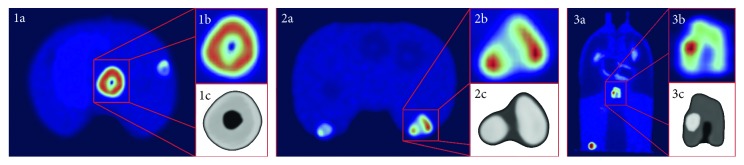
Examples of PET images of heterogeneous lesions (a-b, d-e, g-h), with 3D renders of lesions (c, f, i). (1) *V*_GS_ = 32.3 cc, *S*_GS _= 0.73, *H*_GS_ = 0.16, L/*B*_GS_ = 25; (2) *V*_GS_ = 10.5 cc, *S*_GS_ = 0.62, *H*_GS_ = 0.26, *L*/*B*_GS_ = 10; (3) *V*_GS_ = 8.6 cc, *S*_GS_ = 0.49, *H*_GS_ = 0.25, *L*/*B*_GS_ = 7.

Figure 2 shows 3 PET images of 3 representative heterogeneous lesions including necrosis and bifocal uptake.

### 3.2. Image Segmentation

Table 3 shows the mean percent error on the estimate of MTV for the two considered segmentation methods (adaptive and fixed threshold methods), for small lesions (*V*_GS_ ≤ 10 cc, excluding from *V*_GS_ computation the volume of necrotic regions when present within a lesion), grouped as a function of *L*/*B*_m_ (*L*/*B*_m_ ≤ 5, 5 < *L*/*B*_m_ ≤10, 10 < *L*/*B*_m_ ≤ 15).

The adaptive threshold method presents good results at higher *L*/*B*_m_ (mean percent error <20% for lesion with *L*/*B*_m_ > 5). The percent error of the fixed threshold method is larger (absolute mean percent error >30%), irrespectively from *L*/*B*_m_.

Generally, results show the tendency of the adaptive threshold method to overestimate the volume, while the fixed threshold segmentation method always underestimates it. However, the selection of the optimal segmentation method was not the purpose of this paper.

### 3.3. Radiomic Feature Extraction

Table 4 shows the radiomic features extracted from the segmented MTVs of each lesion.

In particular, five morphological features were extracted characterizing the shape and size of each lesion [8], 13 statistical features were extracted from the analysis of the intensity histogram of lesions, and 40 statistical features were obtained by the textural analysis (9 statistical features for GLCM, 13 for GLRLM, 13 for GLSZM, and 5 for NGTDM), for a total of 58 radiomic features.

### 3.4. Stability of Radiomic Features vs. Segmentation

**Figure 3:**
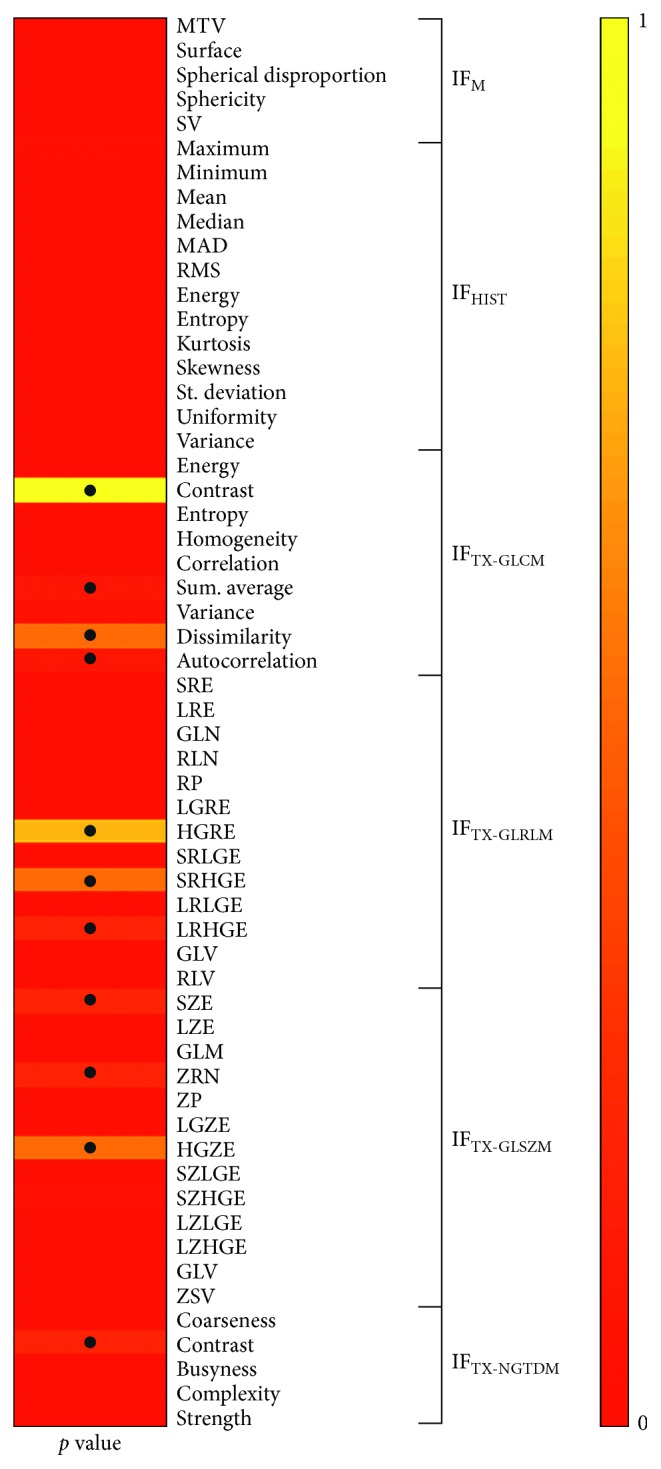
Stability of radiomic features on different segmentations. Friedman test results (*p* value), • indicates *p* value ≥0.05.

Figure 3 shows the results of Friedman test for each of the 58 radiomic features.

By comparing the values of each feature extracted from the MTV as derived from the two segmentation approaches, it was found that many features have a large variability with respect to the applied segmentation method; thus the choice of the segmentation method have a strong impact on the stability of radiomic features.

In particular, results obtained on the whole datasets of both uniform and nonuniform lesions showed that less than 20% (11/58) of radiomic features can be considered full stable with respect the two considered segmentation methods.

**Figure 4:**
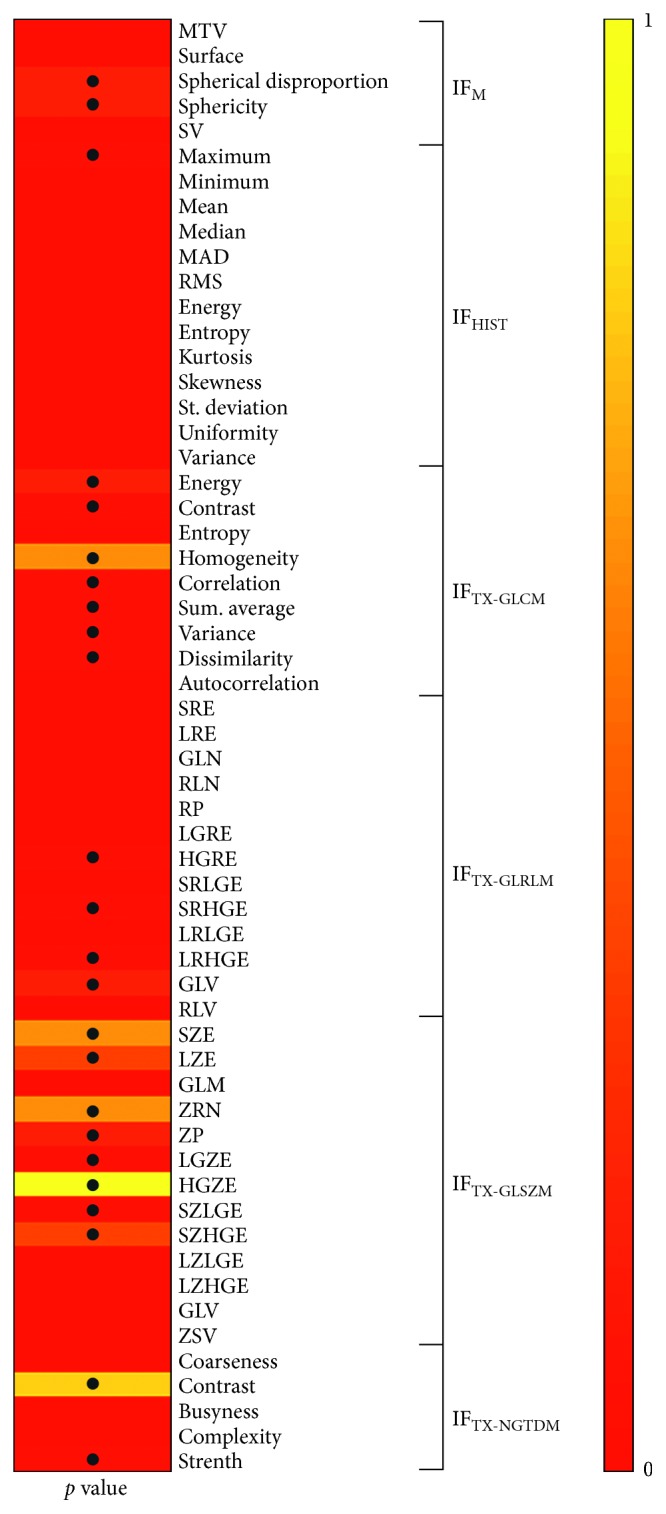
Uniform lesions. Stability of radiomic features on different segmentations. Friedman test results (*p* value), • indicates *p* value ≥0.05.

In Figure 4, the results of Friedman test are presented only for the datasets of uniform lesions.

As expected, a larger number of radiomic features resulted stable (41%, 24/58).

### 3.5. Stability of Radiomic Features vs. Reconstruction

**Figure 5:**
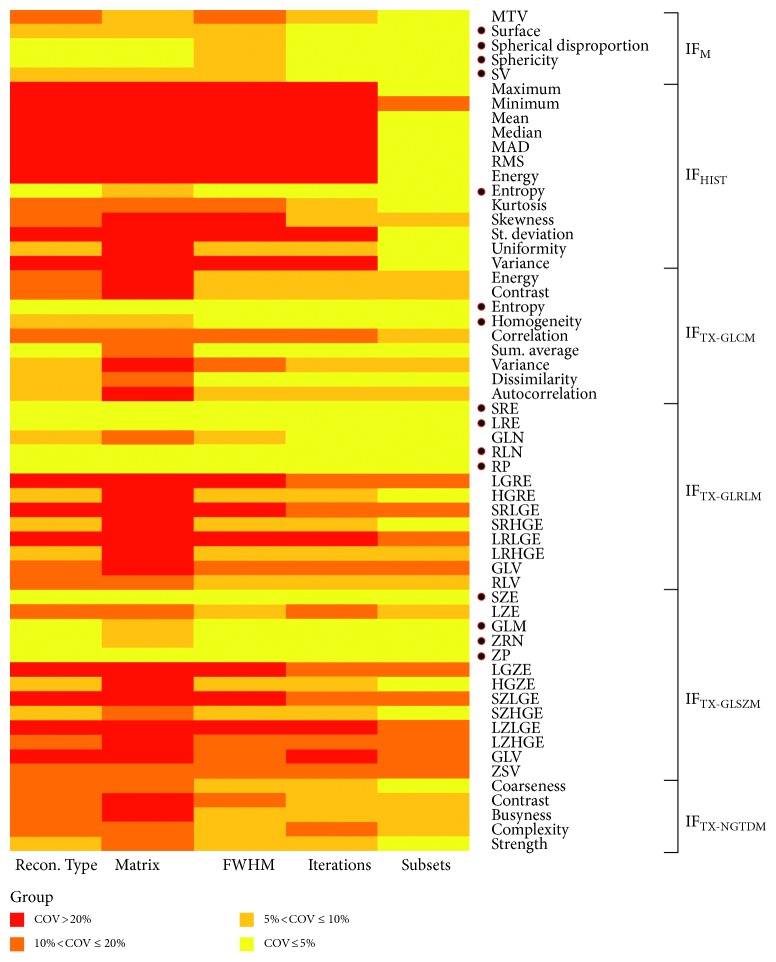
Stability of radiomic features on different reconstruction settings. COV results. • indicates COV ≤ 10%.

Results obtained considering variations of reconstruction parameter (i.e., reconstruction type, matrix size, FWHM of Gaussian filter, number of iterations, and number of subsets) are summarized in Figure 5, grouped with respect to COVs. Reconstruction strongly affects the estimate of radiomic features: 52% (30/58) of features showed a large variability with respect to a different reconstruction setting (COV > 20%). Only 26% (15/58) showed a small variability among all the reconstruction setting variations (COV ≤ 10%). Most features are severely affected by variation in the dimension of reconstructed matrix. Features derived from the analysis of the intensity histogram (IF_HIST_) are more influenced from reconstruction variation than the other features.

### 3.6. Reproducibility of Radiomic Features

**Figure 6:**
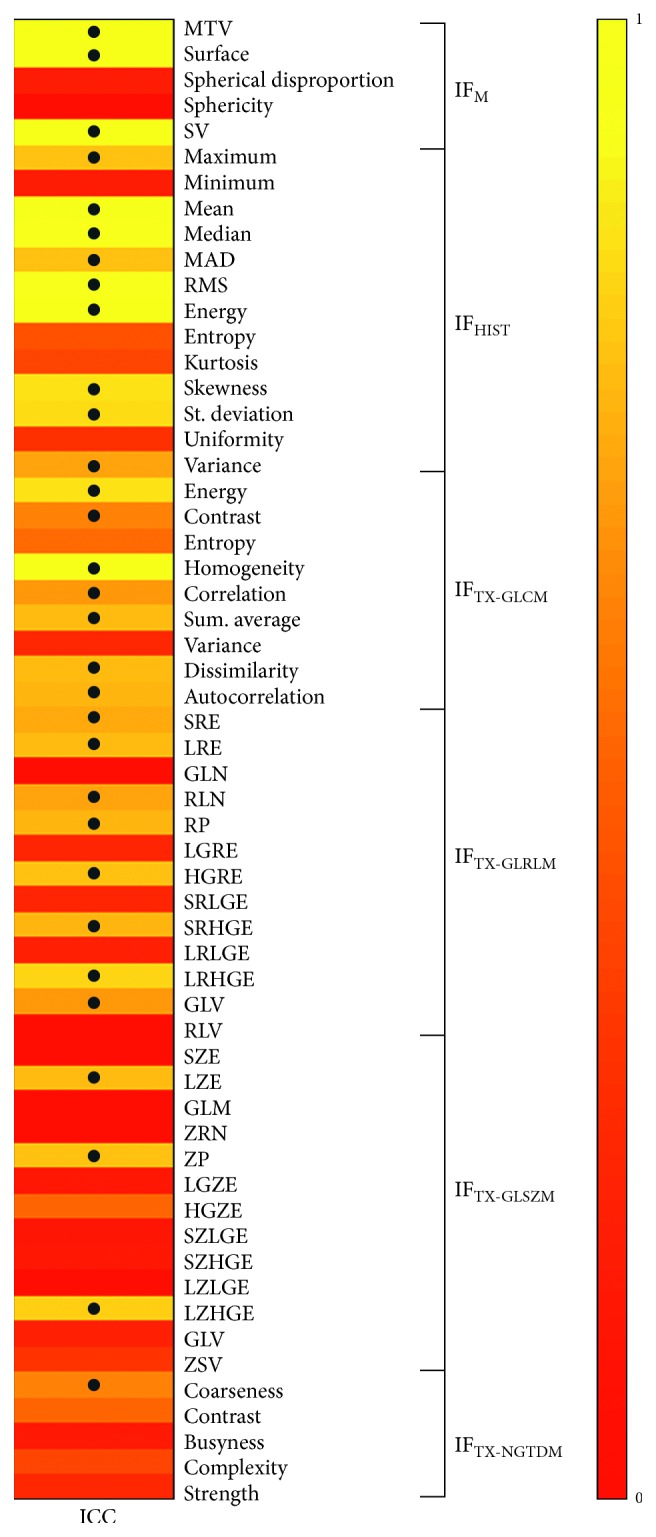
Reproducibility of radiomic features on test-retest datasets. ICC results. • indicates ICC ≥ 0.6.

Thirty-one of the 58 radiomic features (53%) resulted stable in the test-retest datasets (ICC ≥ 0.6), as reported in Figure 6.

### 3.7. Evaluation of Significance of Imaging Features

**Figure 7:**
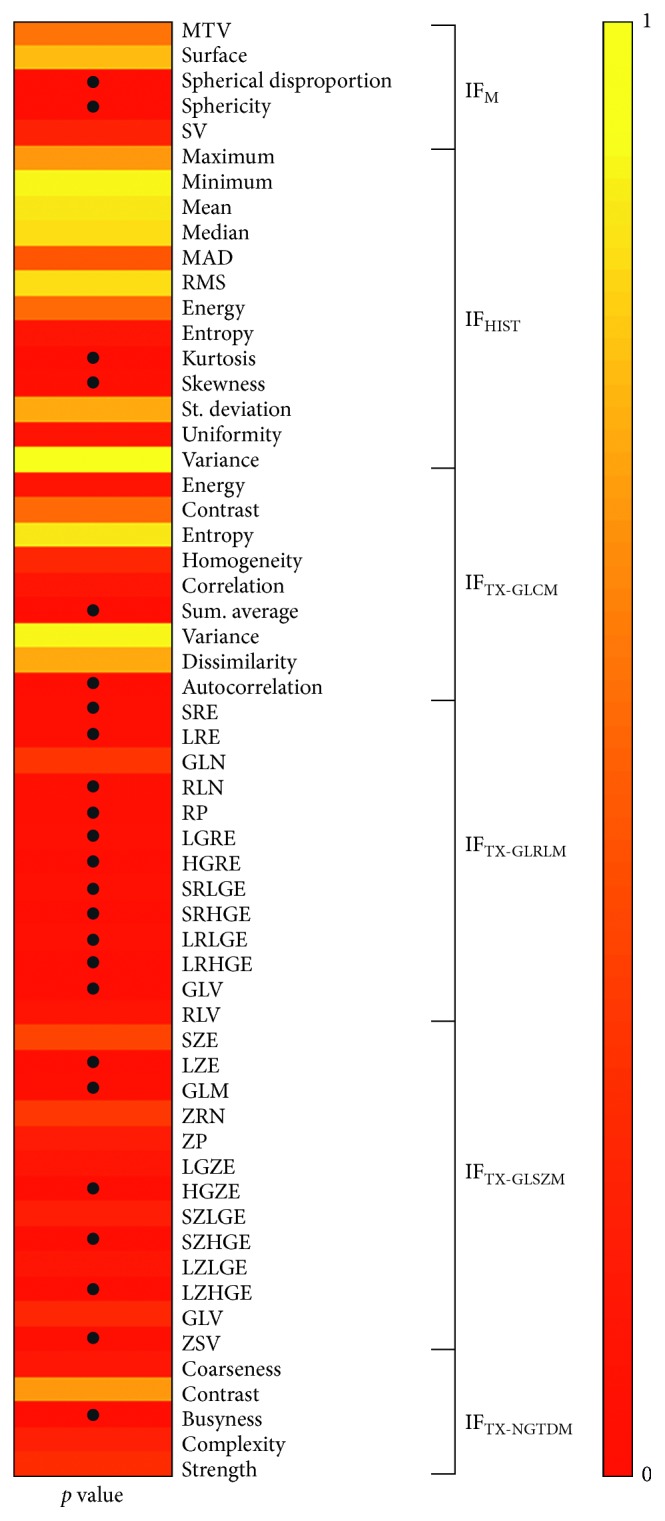
Mann–Whitney test results (*p* value), • indicates *p* value < 0.05.

Results from Mann–Whitney test showed that 24/58 (41%) of radiomic features have significantly different values in case of lesions with uniform versus nonuniform uptake (*p* value < 0.05) (Figure 7).

**Figure 8:**
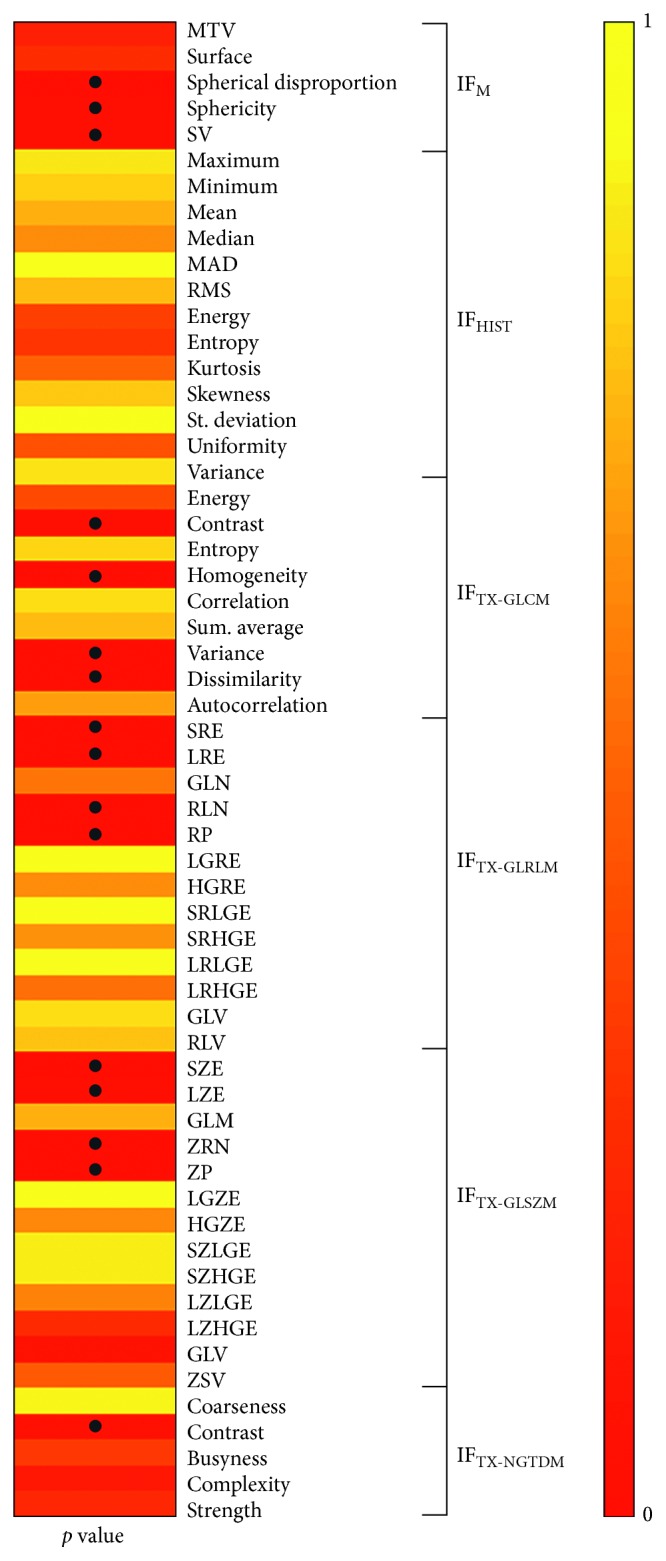
Results of correlation analysis between radiomic features and *H*_GS_ (*p* value), • indicates *p* value < 0.05.

As shown in Figure 8, the correlation analysis performed between radiomic features and *H*_GS_ shows that 16 of them (28%) resulted significantly correlated (*p* value < 0.05).

Paired *t*-test on Gold Standard S_GS_ showed that the morphological IF “Sphericity” is able to reflect actual deviation from spherical shape, both in case of lesions with uniform uptake and in case of lesions with nonuniform uptake.

## 4. Discussion and Conclusions

Despite the potential proven impact of radiomics, scientific evidences suggest that radiomic features extracted from PET images of cancer lesions may have a large variability depending in particular on the different reconstruction settings and segmentation strategies used prior the radiomic analysis [25, 26]. Other studies report that radiomic features can be affected by a lack of intrapatient reproducibility [27–29]. Furthermore, even if the radiomic hypothesis is that such features properly reflect tumor heterogeneity as measured on medical images (including PET images), no clear indications exist concerning which features can better reflect heterogeneous tumor uptake and which type and level of heterogeneity can be captured and quantified through PET.

Some published works were devoted to assess intrapatient reproducibility or features stability with respect to both segmentation or reconstruction settings [11–13]. However, most of these works were performed on patients data or phantom data acquired in ideal conditions (e.g., in spherical synthetic lesions) [12, 30], or on simulated data, where it is difficult to reproduce noise and artifacts contributions, as in real clinical situations [10, 31–33]. Most of these works lack on details about the methodology adopted behind image processing and often evaluate only one aspect of the feature variability [11, 12]. Few works were able to deal with the interpretation of features with respect to tumor heterogeneity and with respect to which type and level of heterogeneity can be quantified through PET [10].

Consistently with other published studies [11, 25, 26, 34, 35], we found that different radiomic PET traits are influenced by the lesion volume delineation method (less than 20% features can be considered stable for the two methods assessed in this work), our results confirming that the choice of segmentation method severely affects the quantitative estimate of radiomic features. Such concern regards in particular the possibility to compare results obtained by radiomic studies in which different segmentation methods were used, as occurring in some multicenter evaluations proposing databases of reconstructed images with lesions segmented by operators and annotated on the archived images. To avoid bias in the results, our findings suggest the use of the same segmentation method to be applied with a standardized image processing procedure, possibly with the use of the same software tools, after image collection and archive.

The fixed threshold approach is widely used in the literature [12], and for this reason, we have used this method to segment the lesion volume. However, notwithstanding this was not the purpose of our paper, our results are in agreement with many published studies, showing that the accuracy in the definition of lesion volume is low [13]. In particular, the method risks to largely overestimate the lesion volume (large and negative percent errors), and this, in addition to a poor accuracy, can cause severe problems in the estimate of radiomic features due to the possible inclusion of signal uptake not linked to cancer tissue but to the surrounding tissues. The adaptive threshold approach seems to be more suitable for radiomic analysis since it is more conservative with respect to the estimate of cancer tissue volume.

We found, in agreement with previous reports [11] that also reconstruction largely affects the estimate of radiomic features (only 26% are stable with respect to different settings). In particular, the more impacting parameter is the reconstructed matrix size that leads to variations in the estimate of many radiomic features greater than 20%. To avoid bias in the results, similarly to what suggested as segmentation strategy, our findings would suggest the use of the same reconstruction method to be applied with the same image reconstruction setting, possibly with the use of the same reconstruction tool, after the acquisition and archive of raw data. Unfortunately, while a high level of standardization is possible for the segmentation step, this is difficult for the reconstruction step, for different reasons. Clinical images are reconstructed with different reconstruction algorithms depending from the physical characteristics of the imaging systems/models and various reconstruction settings defined in different imaging centers. This limits the possibility to standardize the reconstruction protocol in prospective clinical studies or to have access to retrospective studies with the same reconstruction protocols used. The only way to perform valid radiomic studies should be to collect raw data from prospective patient studies and then to reconstruct them with the same reconstruction tool, but this is a very challenge task to be accomplished, in particular for the huge amount of resources (in space and time) required. Orlhac et al. [10] very recently have proposed a method based on the ComBat approach [36] used in genomics analysis that seems effective in standardizing radiomic features measured from PET images obtained using different imaging protocols.

Intrapatient reproducibility can be a serious concern, but it could be properly managed. A good number of features (31) resulted reproducible from our results of test-retest setting, suggesting to consider this subset for further radiomic analysis. Among these reproducible features we found most of morphological and histogram-derived features considered in this work, and some textural features from the gray-level co-occurrence matrix and gray-level run-length matrix.

Eleven of the 31 features were found also able to discriminate heterogeneous from uniform radioactivity uptake (*p* value from Mann–Whitney test <0.05).

Furthermore, interesting results were obtained when comparing radiomic features with respect to gold standard indexes of heterogeneity and sphericity. Considering the uptake heterogeneity, we found 3 reproducible features (run-length-nonuniformity, run percentage, and large zone emphasis) among the 11 found above, which are also proven able to reflect the heterogeneity in the PET uptake (strongly correlated with the gold standard heterogeneity index). These findings suggest that the 3 features can be considered as first choice when testing the hypothesis that PET heterogeneity could reflect real tumor heterogeneity.

In conclusions, in this work, we showed some limits and quantitative properties of the radiomics approach (with respect of the tested methods and parameters) that should be overcome for a clinical translation of radiomics. Considering our findings, we suggest an optimal strategy for radiomic bias-free analysis to archive all raw data of PET acquisitions collected for a clinical study, to be then reconstructed and segmented by standardized reconstruction and segmentation protocols. We found a subset of thirty features that could be preferred for reproducible radiomic PET studies; 3 of them seeming particularly suitable for capturing tumor heterogeneity. However, our results need to be confirmed by other more extensive studies and cannot be exactly transferred to real or more complex clinical conditions.

## Figures and Tables

**Table 1:** Reconstruction settings.

Reconstruction algorithm		Number of iterations	Number of subsets	FWHM Gaussian filter (mm)	Reconstructed matrix size
Impact of reconstruction algorithm	OSEM3D	3	18	5	256
	OSEM3D + PSF				
	OSEM3D + TOF				
	OSEM3D + PSF + TOF				
Impact of number of iterations	OSEM3D + PSF + TOF	2	18	5	256
		3			
		4			
Impact of number of subsets	OSEM3D + PSF + TOF	3	18	5	256
			24		
Impact of reconstructed matrix size	OSEM3D + PSF + TOF	3	18	5	128
					192
					256
Impact of FWHM of Gaussian filter	OSEM3D + PSF + TOF	3	18	5	192
				7	

OSEM = ordered subset expectation maximization; PSF = point spread functions; TOF = time of flight; FWHM = full width at half maximum.

**Table 2:** Summary of the 18F-FDG PET/CT acquisitions of the phantom, with gold standard values of each lesion.

Number of PET acquisition	Number of lesion acquired in PET	Shell type	*V*_GS_ (cc)	*S*_GS_	*H*_GS_ (%)	*V*_GS_ excluding necrosis (cc)	*L*/*B*_GS_
1	1	A	6.8	0.57	0	6.8	10
	2	B	10.5	0.62	0	10.5	10
	3	C	8.5	0.49	0	8.5	10
	4	D	12.5	0.74	0	12.5	10

2	5	A	6.8	0.57	0	6.8	10
	6	B	10.5	0.62	0	10.5	10
	7	C	8.5	0.49	0	8.5	10
	8	D	12.5	0.74	0	12.5	10

3	9	A	6.8	0.57	0	6.8	10
	10	B	10.5	0.62	0	10.5	10
	11	C	8.5	0.49	0	8.5	10
	12	D	12.5	0.74	0	12.5	10

4	13	A	6.8	0.57	0	6.8	27
	14	B	10.5	0.62	0	10.5	26
	15	C	8.5	0.49	21.1	7.4	9
	16	D	12.5	0.74	12.7	11.7	25

5	17	A	6.8	0.57	0	6.8	27
	18	B	10.5	0.62	0	10.5	26
	19	C	8.5	0.49	21.1	7.4	9
	20	D	12.5	0.74	12.7	11.7	25

6	21	A	6.8	0.57	0	6.8	27
	22	B	10.5	0.62	0	10.5	26
	23	C	8.5	0.49	21.1	7.4	9
	24	D	12.5	0.74	12.7	11.7	25

7	25	A	6.8	0.57	0	6.8	12
	26	B	10.5	0.62	0	10.5	11
	27	C	8.5	0.49	21.1	7.4	4
	28	D	12.5	0.74	12.7	11.7	11

8	29	A	6.8	0.57	14.9	6.4	18
	30	B	10.5	0.62	26.2	10.5	10
	31	C	8.5	0.49	24.8	7.6	7
	32	D	12.5	0.74	62.2	8.7	9
	33	E	32.3	0.73	16.3	29.4	25

9	34	A	6.8	0.57	14.9	6.4	12
	35	B	10.5	0.62	26.2	10.5	7
	36	C	8.5	0.49	24.8	7.6	5
	37	D	12.5	0.74	62.2	8.7	6
	38	E	32.3	0.73	16.3	29.4	16

**Table 3:** Mean percent error on the estimate of MTV of small lesions as a function of *L*/*B*_m_, for the adaptive and fixed threshold segmentation methods.

*L*/*B*_m_	Adaptive threshold mean percent error (%)	Fixed threshold mean percent error (%)
*L*/*B*_m _≤ 5	27 ± 9	−33 ± 13
5 < *L*/*B*_m_ ≤ 10	16 ± 30	−35 ± 26
10 < *L*/*B*_m_ ≤ 15	17 ± 16	−31 ± 25

**Table 4:** The radiomic features considered in the work.

Feature name	Feature group
MTV	IF_M_
Surface	
Spherical disproportion	
Sphericity	
Surface-volume ratio (SV)	

Maximum	IF_HIST_
Minimum	
Mean	
Median	
Mean absolute deviation (MAD)	
Root mean square (RMS)	
Energy	
Entropy	
Kurtosis	
Skewness	
Standard deviation	
Uniformity	
Variance	

Energy	IF_TX-GLCM_
Contrast	
Entropy	
Homogeneity	
Correlation	
SumAverage	
Variance	
Dissimilarity	
Autocorrelation	

Short run emphasis (SRE)	IF_TX-GLRLM_
Long run emphasis (LRE)	
Gray-level nonuniformity (GLN)	
Run-length nonuniformity (RLN)	
Run percentage (RP)	
Low gray-level run emphasis (LGRE)	
High gray-level run emphasis (HGRE)	
Short run low gray-level emphasis (SRLGE)	
Short run high gray-level emphasis (SRHGE)	
Long run low gray-level emphasis (LRLGE)	
Long run high gray-level emphasis (LRHGE)	
Gray-level variance (GLV)	
Run-length variance (RLV)	

Small zone emphasis (SZE)	IF_TX-GLSZM_
Large zone emphasis (LZE)	
Gray-level nonuniformity (GLN)	
Zone-size nonuniformity (ZSN)	
Zone percentage (ZP)	
Low gray-level zone emphasis (LGZE)	
High gray-level zone emphasis (HGZE)	
Small zone low gray-level emphasis (SZLGE)	
Small zone high gray-level emphasis (SZHGE)	
Large zone low gray-level emphasis (LZLGE)	
Large zone high gray-level emphasis (LZHGE)	
Gray-level variance (GLV)	
Zone-size variance (ZSV)	

Coarseness	IF_TX-NGTDM_
Contrast	
Busyness	
Complexity	
Strength	

## Data Availability

An image set of our original anthropomorphic phantom is available to researchers, after registration, at http://inlab.ibfm.cnr.it/inlab/research_data.php.
